# Quality of life in phenylketonuria (PKU) patients residing in Iehran, Islamic republic of Iran

**Published:** 2015

**Authors:** H Hatami, S Khodakarim, A Sotoodeh, A Nabizadeh, R Radfar

**Affiliations:** * Department of Public Health and Environmental and Occupational Research Center, School of Health, Shahid Beheshti University of Medical Science, Tehran, Iran; ** Department of Epidemiology, School of Health, Shahid Beheshti University of Medical Science, Tehran, Iran; *** Pediatric Endocrinology, Tehran University of Medical Center, Tehran, Iran; **** Pharmacoeconomics & Pharmaceutical Administration, Tehran University, Tehran, Iran; ***** Agri-industries Research Department, Agricultural planning, Economic and Development Research Institute, Tehran, Iran

**Keywords:** quality of life, social health, phenylketonuria (PKU) patients, WHOQOL BREF questionnaire

## Abstract

**Background:** The current study tried to evaluate the quality of life (QOL) of phenylketonuria (PKU) patients residing in Tehran, Iran and it also tried to determine the average quality of life of patients. Various aspects of QOL have been analyzed depending on gender, age, and educational levels of the subjects.

**Methods:** The sample of the study consisted of late-diagnosed PKU patients who were referred to Mofid Children’s Medical Center as well as to Ali-Asghar Hospitals in order to receive metabolic diets on a one year period starting from spring 2013 to spring 2014. Due to the limited study population, subjects were selected via census, therefore 82 patients were enrolled. The research material consisted of the Persian edition of World Health Organization Quality of Life questionnaire (WHOQOL-BREF), designed to examine physical, mental, social and environmental health. The data was gathered on two levels-descriptive and inferential- by using the SPSS software, V.20.

**Results:** Results indicated that the low quality of life in the late-diagnosed patients suffering from PKU, with mental, physical, social, and environmental aspects, was below the average. Still, even if it was not gender dependent, QOL was greatly influenced by the educational level of the patients. Moreover, it was discovered that the mental health of the patients above 40 years old was significantly lower than the other age groups.

**Conclusions:** According to the findings of this study, it was recommended that special attention should be given to the improvement of the social and mental health of PKU patients.

## Introduction

Phenylketonuria is the most commonly amino acid metabolism disorder, as well as the primary cause of mental retardation. Inherited as an autosomal recessive disease and considered a defect in the hepatic enzyme, Phenylalanine hydroxylase is the primary causative factor in its pathogenesis [**1**-**3**].

PKU is being screened in the United States of America and many European countries since 1960 [**4**]. Newborn screening for classic PKU started in Iran in 2007 and has extended to the now-classical PKU, since 2010. The objectives of the screening were 1) to identify the newborns with PKU as soon as possible and decrease the mortality and morbidity and to reduce the mental retardation; 2) to identify the prenatal diagnosis in the families with an index patient with PKU, and 3) to improve the control and quality of life in those patients with late diagnosed cases of PKU, even if they were referred to selected centers in the university hospitals around the country. For example, in Tehran, they were referred to Mofid, Children’s Medical Center and Aliasghar hospitals [**4**-**6**].

According to WHO, quality of life is defined as the understanding of the individuals’ living status from a cultural perspective and the determination of the dominant values of each population. As the concept implies, quality of life is a personal concept based on the values of every person but incomprehensive to others [**7**-**9**].

Although the quality of life is considered a subjective issue by the WHO, Philips analyzed this concept from various individual and social approaches, based on both subjective and objective parameters. In Philips’ opinion, it is necessary to meet the social demands of basic needs and provide financial and materialistic resources to achieve the objective values of QOL [**10**] However, it is necessary to achieve satisfaction, gratification, personal and intellectual growth and spiritual blooming as well as altruism and most significantly social engagement at the highest levels. From a social approach, QOL signifies the importance of a stable physical and social environment, social resources within the groups and communities in which the individuals take part in, such as civilized integrity, complex social networks, synergism and integrity and values such as altruism, social justice, fairness, and equality [**11**].

Researchers have suggested various indexes to assess QOL. **Table 1** illustrates the QOL parameters and some comparisons between them.

**Table 1:** Experimental QOL assessment indexes from various approaches

PhelsandPeri (1993)	Shalaque (2000)	WHO (1993)	Hagarty et al. (2001)	Camins (1997)
Financial wellbeing	Financial wellbeing	Financial wellbeing	Health	Health
Physical wellbeing	Physical wellbeing	Environment	Financial wellbeing	Financial wellbeing
Social wellbeing	Social liability	Social relationships	Sense of social acceptance	Social wellbeing
Productive health	Emotional health	Psychological	Productivity	Productivity
Emotional health	Rights	Independence level	Emotional health	Emotional health
Civil health	Interpersonal connections	Spiritual health	Friendships and familiar connections	Social and familiar connections
-----------	Personal improvement	-----------	Personal security	Security
-----------	Independence	-----------	-----------	-----------

The increasing popularity of QOL as a complex term has led to an increase in the fields covering this concept. In his studies, Hacker suggested the physical, psychological and social domains of QOL for cancer patients [**12**]. On the other hand, Ozalp stated that the aspects of social, economic and environmental aspects are the three main pillars of QOL. It is believed that these factors may help in understanding the significance of an appropriate and stable life as well as the quality of life [**13**].

 The current study evaluated the QOL of PKU patients residing in Tehran. It also tried to analyze the quality of life of patients according to age, gender, and education. 

## Methods 

**Study population**

The study population consisted of late-diagnosed PKU patients who had been hospitalized in Mofid, Children’s Medical Center, and Ali Asghar hospitals. The PKU patients were required to have an intellectual level, which enabled them to answer to the items of the questionnaire. The study was conducted between spring 2013 and spring 2014.

**QOL assessment**

The research tool consisted of the Persian edition of the Quality of Life questionnaire provided by WHO (WHOQOL-BREF), which was answered during interviews. The survey was designed to assess the QOL from four main perspectives, as it follows physical, psychological, social, and environmental health. The questions listed were answered in grades, one being the lowest level and five being the highest level. In the end, the higher the overall score, the better the QOL. The data were collected and analyzed by using SPSS software V.20. 

**Statistical analysis**

The descriptive analysis was reported in the shape of medians, standard deviations, and frequencies. The objective analysis was presented in the form of meaningful differences in QOL and its varying aspects according to age, gender, and educational factors. Moreover, the T-tests and variance analysis were employed. 

The distribution analysis showed that 42.7% of the samples were men, and 57.3% were women; 17.1% were illiterate, 63.5% either had a diploma or were educated up to lower levels and 19.5% received higher education. Moreover, the distribution analysis showed that 41.5% were younger than 20 years old, 48.8% were between 20 and 29 years old, 4.9% were between 30 and 39 years old, and another 4.9% were older than 40. In terms of the time of the diagnosis of PKU in patients, 29.3% were diagnosed between 7 and 15 months, 41.5% were diagnosed between 16 and 24 months, 24.4% were diagnosed between 25 and 40 months and the remaining 4.9% were diagnosed with PKU in more than 40 months (between 1 to 180 months). The median duration from the diagnosis was determined to be of 32 months, with a standard deviation of 4.60. The rate of consanguineous marriage among the PKU families was 85.4%.

## Results 

In this section, a description of the QOL of PKU patients and its various aspects were defined in comparison to the theories previously mentioned by using the corresponding parameters. 

**Table 2** illustrates the median values, standard deviation, minimum and maximum QOL of PKU patients who were categorized according to three parameters.

**Table 2:** Descriptive indexes of QOL in PKU patients with main parameters

QOL and pertaining aspects	Range	Median value	Standard deviation	Minimum	Maximum
QOL (overall score)	1-5	2.60	0.505	1.44	3.96
Physical health	1-5	2.83	0.524	1.71	4.43
Psychological health	1-5	2.82	0.696	1.17	4.50
Social connections	1-5	2.37	0.763	1.00	4.07
Environmental health	1-5	2.37	0.533	1.13	3.63
QOL= Quality of Life					

The results listed in **Table 2** showed that phenylketonuria patients had an overall QOL, as well as its pertaining aspects, which were considerably lower than average. Amongst these, physical and psychological health had higher values, while environmental and social connections were significantly lower. In general, the lowest QOL determined was 1.44 and the highest was 3.96. In the following section, the three theories, which were discussed earlier, were used to determine the significance level of the difference between QOL aspects based on the gender of the subjects, education, age and the time elapsed from the primary diagnosis. 

Before employing the T-test, the regular distribution pattern of the data was confirmed by using the Kolmogorov–Smirnov test. Therefore, the results showed that the significance level of the KS test was above 0.05, confirming the normality of the provided data. **Table 3** presents the importance of the variation between the average QOL of the subjects and its pertaining attributes, based on gender.

**Table 3:** Comparing the average QOL and its pertaining aspects based on the gender, in PKU patients

Variable		Frequency	Average	Degree of freedom	T-test	Degree of significance
QOL	Men	32	2.56	72	-0.701	0.485
	Women	42	2.63			
Physical health	Men	32	2.83	72	0.185	0.854
	Women	42				
Psychological health	Men	32	2.75	72	-0.346	0.743
	Women	42				
Social health	Men	32	2.27	72	-1.474	0.145
	Women	42				
Environmental health	Men	32	2.38	72	-0.103	0.918
	Women	42				
QOL= Quality of Life						

The results of **Table 3** showed that no significant variation was present between the QOL of PKU patients and its pertaining aspects between genders. The QOL in both men and women was roughly the same, and both genders experienced a similar quality of life. 

**Table 4:** Comparing the average QOL in PKU patients and its pertaining attributes based on the education received by the subjects

Variable		Sum of squares	Degree of freedom	Average of square values	F- test	Degree of significance
Regression of remaining total	QOL	4.862	3	1.609	7.889	0.000
		15.906	78	0.204		
		20.737	81			
Regression of remaining total	Physical health	2.942	3	0.981	3.961	0.011
		19.312	78	0.248		
		22.254	81			
Regression of remaining total	Psychological health	9.478	3	3.159	8.266	0.000
		29.813	78	0.513		
		39.293	81			
Regression of remaining total	Social health	5.312	3	1.177	3.298	0.025
		41.873	78	0.513		
		47.184	81			
Regression of remaining total	Environmental social	3.936	3	1.312	5.364	0.002
		19.080	78	0.245		
		23.016	81			
QOL= Quality of Life						

The results of **Table 4** showed that a significant variation was present between the QOL of PKU patients and its pertaining aspects, when studied from an educational perspective. Those patients who received higher education experienced a higher quality life when compared to those who did not. To determine the primary cause of such differences, the LSD pursuit test was employed. Results showed that when comparing the General QOL of PKU patients or even its varying aspects, individuals who managed to obtain their diploma or reached higher degrees of scientific excellence, also tended to experience a higher quality of life, which was significantly different from the life of those who were illiterate. It may be said that education played a key role in the understanding that PKU patients have their lives and a quality at which they experience it.

**Table 5:** Comparing the average QOL in PKU patients and its pertaining attributes based on the age group of the subjects

Variable		Sum of square values	Degree of freedom	Average of square values	F- test	Degree of significance
Regression of remaining total	QOL	0.963	3	0.321	1.266	0.292
		19.769	78	0.253		
		20.732	81			
Regression of remaining total	Physical health	0.512	3	0.171	0.612	0.609
		21.742	78	0.279		
		22.254	81			
Regression of remaining total	Psychological health	0.953	3	1.984	4.642	0.005
		33.339	78	0.427		
		39.292	81			
Regression of remaining total	Social health	1.853	3	0.618	1.063	0.370
		45.332	78	0.581		
		47.184	81			
Regression of remaining total	Environmental social	0.081	3	0.027	0.091	0.965
		22.935	78	0.294		
		23.016	81			
QOL= Quality of Life						

**Table 5** presented the results of the analysis performed in order to compare the degree of significance between the four age groups. It was obvious that the QOL of the subjects and its pertaining aspects were not significantly affected by the age of the subjects. The patients in different ages experienced a similar state of physical, mental, social, and environmental health. However, the LSD pursuit test revealed that patients who were older than 40, varied from other age groups in the parameter of psychological health; the level of mental health, in patients above 40 years old being in a poorer state when compared to the younger age groups. 

## Discussion 

The current study tried to evaluate the quality of life of late-diagnosed phenylketonuria (PKU) patients residing in Tehran, Iran. The results revealed that patients tended to experience a lower physical, psychological, social, and environmental health than the general population. It was also shown that even though gender had no significant impact on the value of the above parameters, the indexes were significantly affected by the age and the educational level of the patients. Those patients who received a higher education had a better QOL and patients who were above the age of 40, had a lower index. 

Various studies, such as Cazorla et al.’s, reached the conclusion that QOL is significantly weaker in those who suffer from phenylketonuria than in the general population. Also, these patients experience lower physical, psychological, social and environmental health [**1**]. Bosch showed that the QOL of the patients who were diagnosed and treated in early stages is roughly equal to that of normal and healthy people [**11**]. Eva Simon et al. revealed that when treated in the primary stages of the illness, the patient might be neurologically fit to benefit from an intellectual level [**14**]. These findings emphasized the importance of neonatal PKU screening, which has been performed in Iran since 2010. However, the subjects of the sample were born before the nationalization of PKU screening and could not have been treated sooner or in the earlier stages. The result showed that these patients experienced a QOL lower than average. 

Further, the findings of this study indicated that individuals who received higher levels of education also experienced a higher QOL [**11**,**13**,**15**], these results being in accordance with the findings of Cazorla et al.[**1**]. This may be because higher education provides better occupation and more suitable life conditions, thus a higher QOL.

Phenylketonuria has a relatively high prevalence in Iran (1 in 4800 live births), which may be due to the high percentage of consanguineous marriages [**16**], making it necessary for prenatal diagnosis [**17**]. Access to small protein products in Iran is limited, and a big challenge as the diet is lifelong [**15**,**18**], therefore the patients should be encouraged, and psychological support should be provided through the PKU clinics. It seems that the National PKU screening will provide a big chance to diagnose the PKU patients very early in life, so as to start the special diet and to prepare the family and the patient for a lifelong disease [**14**,**19**]. It is possible to have alternative options to increase the phenylalanine allowance in the food and to make it affordable for the patients. One of these approaches is using sapropterin hydrochloride (Kuban) tablets in patients with mild to moderate PKU. In a study in Tehran, 90% of the mild hyperphenylalaninemia and PKU patients and 35.7% of the moderate PKU responded to Kuvan either by getting off the diet or by increasing the phenylalanine tolerance. This approach can improve the QOL in these patients [**20**,**21**]. 

This paper was taken from the MPH thesis that was conducted in the Faculty of Public Health–Shahid Beheshti University.
